# Temporal trends and adverse perinatal outcomes of twin pregnancies at differing gestational ages: an observational study from China between 2012–2020

**DOI:** 10.1186/s12884-022-04766-0

**Published:** 2022-06-03

**Authors:** Peiran Chen, Mingrong Li, Yi Mu, Yanping Wang, Zheng Liu, Qi Li, Xiaohong Li, Li Dai, Yanxia Xie, Juan Liang, Jun Zhu

**Affiliations:** 1 National Office for Maternal and Child Health Surveillance of China, West China Second University Hospital, Sichuan University, Chengdu, Sichuan China; 2 Medical Big Data Center, Sichuan University, Chengdu, Sichuan China; 3 Department of Obstetrics, West China Second University Hospital, Sichuan University, Chengdu, Sichuan China; 4 Key Laboratory of Birth Defects and Related Diseases of Women and Children (Sichuan University), Ministry of Education, Chengdu, Sichuan China

**Keywords:** Twins, Trend, Stillbirth, SGA, Low apgar score, Gestational age

## Abstract

**Background:**

With the development of assisted reproductive technology, the twinning rate in China has been increasing. However, little is known about twinning from 2014 onwards. In addition, previous studies analysing optimal gestational times have rarely considered maternal health conditions. Therefore, whether maternal health conditions affect the optimal gestational time remains unclear.

**Methods:**

Data of women delivered between January 2012 and December 2020 were collected through China’s National Maternal Near Miss Surveillance System. Interrupted time series analysis was used to determine the rates of twinning, stillbirth, smaller than gestational age (SGA), and low Apgar scores (< 4) among twins in China. To estimate the risk of each adverse perinatal outcome for separate gestational weeks, a multivariate generalised linear model was used. Infants born at 37 weeks of gestational age or foetuses staying in utero were used as reference separately. The analyses were adjusted for the sampling distribution of the population and the cluster effect at the hospital and individual levels were considered.

**Results:**

There were 442,268 infants enrolled in this study, and the adjusted rates for twinning, stillbirth, SGA, and low Apgar scores were 3.10%, 1.75%, 7.70%, and 0.79%, respectively. From 2012 to 2020, the twinning rate showed an increasing trend. Adverse perinatal outcomes, including stillbirth, SGA, and low Apgar scores showed a decreasing trend. A gestational age between 34 and 36 weeks decreased most for rate of stillbirth (average changing rate -9.72%, 95% confidence interval [CI] -11.41% to -8.00%); and a gestational age of between 37 and 38 weeks decreased most for rates of SGA (average changing rate -4.64%, 95% CI -5.42% to -3.85%) and low Apgar scores (average changing rate -17.61%, 95% CI -21.73% to -13.26%). No significant difference in changes in twinning rate or changes of each perinatal outcome was observed during periods of different fertility policies. Infants born at 37 weeks of gestation had a decreased risk of stillbirth, SGA, and low Apgar scores. Maternal antepartum or medical complications increased the risk of SGA and low Apgar scores in different gestational weeks.

**Conclusion:**

China’s twinning rate showed an increasing trend, while adverse perinatal outcomes decreased from 2012 to 2020. Fertility policy changes have had little effect on the twinning rate or the rate of adverse perinatal outcomes such as stillbirth, SGA, or low Apgar scores. The optimal gestational age for twins was 37 weeks. Women pregnant with twins and with antepartum or medical complications should be cautious due to an increased risk of SGA and low Apgar scores.

**Supplementary Information:**

The online version contains supplementary material available at 10.1186/s12884-022-04766-0.

## Introduction

Since the 1970s, twin births have increased rapidly in developed countries [1]. However, due to an insufficient registration system, the condition of twins is rarely reported in less developed countries. Xinrong Lu et al. reported that the twinning rate in southeast China, part of one of the largest developing countries, has remained constant during 1993–2005 [2]. Deng et al. reported an increasing trend in the Chinese twinning rate from 2007 to 2014 [3]. However, little is known about the trend in twins after 2014. Research has shown that changes in the Fertility policy in 2016, namely, the introduction of the “universal two child policy”, have had an effect on the number of new-born infants. There were 5.4 million new-borns promoted by the new policy, but this change seemed to have little effect on the outcomes of perinatal health [4]. Whether the policy change has had an effect on twin births remains unknown.

Evidence has shown that the relative risk of adverse outcomes may depend on gestational age [5, 6]. Women with a twin pregnancy are more likely to undergo preterm birth, and approximately only half of women will give birth after 37 weeks of gestation [7]. Thus, the optimal gestational age for twin pregnancies may differ from that of singletons. The United Kingdom (UK) clinical guidelines support a policy of elective delivery from 37 weeks 0 days in dichorionic pregnancies (two placentae and two separate chorions) and 36 weeks 0 days in monochorionic pregnancies (one placenta and either one or two chorions) in order to reduce adverse short-term outcomes in twins, such as perinatal mortality [8]. Previous multiple population-based studies have indicated that births delivered between 37 and 39 weeks had the lowest risk of perinatal mortality and morbidity [9, 10]. However, these studies rarely took maternal and perinatal complications into consideration and concluded the “optimal gestational delivery week” specifically on “women with an uncomplicated twin pregnancy” [11, 12]. Whether maternal complications can have an effect on the recommended optimal gestational week is largely unknown. On the other hand, a significant number of twin-analysis research has suffered from small sample sizes and low twinning birth rates, making it difficult to accumulate a large set of twin data. This study, based on China’s National Maternal Near Miss Surveillance System (NMNMSS), collected data from 2012 to 2020, covering 30 provinces of mainland China. The system has helped in policy development and disease burden assessments in both China and worldwide [13–15]. The number of samples collected by this system is sufficient for us to determine the twinning rate and rate of each adverse perinatal outcome after the introduction of the new fertility policy, and to explore the optimal gestational weeks for twin pregnancies with the fewest adverse neonatal outcomes among women with different health conditions.

## Methods

### Data collection

Individual data regarding women who delivered between January 2012 and December 2020 were collected through China’s NMNMSS. The NMNMSS system covers 441 member hospitals that manage more than 1000 deliveries annually. The included member hospitals are located in 326 districts or counties throughout 30 provinces in mainland China, excluding Tibet. All collected data were quality controlled at the provincial, municipal, and county levels at least four times annually [16]. The NMNMSS collects the sociodemographic, obstetric, and perinatal information of pregnant and postpartum women from the Obstetric Departments of surveillance hospitals. The data collected for this study included the name and code of the hospital, date of delivery, number of antenatal visits, maternal educational and marital status, maternal age, delivery mode, foetal sex, parity, and number of foetuses. The sampling strategy, data collection, and quality control procedures have been detailed elsewhere [14, 16, 17].

### Definitions

The twinning rate was defined as twin-born infants among all infants born after 28 weeks of gestational age. Adverse perinatal outcomes included stillbirth, smaller than gestational age (SGA), and low Apgar scores (< 4). Stillbirth was restricted to births that occurred after 28 or more weeks of gestation. Gestational age in China is generally ascertained on the basis of the last menstrual period or ultrasound examination when the date of the last menstrual period is unknown [17]. SGA was defined as occurring when infants’ birthweights were less than the 10^th^ percentile for gestational age, in reference to the population-based birth weight for Chinese twins [18]. A low Apgar score was defined as an Apgar score of less than 4 measured at 1 min after birth.

Weight discordance among twins, a covariate, was defined as (larger weight smaller weight)/larger weight*100%) ≥ 20% [19]. Maternal complications were defined as mutually exclusive categories including antepartum complications, medical diseases, and none of the above. If women had both antepartum complications and medical diseases, they were categorised as part of the antepartum complications group. Antepartum complications included ruptured uterus, placenta previa, abruptio placentae, unspecified antepartum haemorrhage, hypertensive disorders, or any foetal malpresentation (breech, shoulder, or other). Medical diseases included heart disease, embolism or thrombophlebitis, hepatic disease, severe anaemia (haemoglobin concentration < 70 g/L), renal disease (including urinary tract infection), lung disease (including upper respiratory tract infection), AIDS, connective tissue disorders, gestational diabetes, and cancer [20]. Based on the hospitals’ location, we classified the areas as urban or rural, and geographic location as east, central, or west. The hospital level (from Level 1 to Level 3) was certified by the Administrative Department of Health according to the number of beds, categories of clinical departments, number of medical personnel, type and quantity of equipment, and hospital funding; level 3 hospitals are considered to have more advanced care. Other variables, including prenatal examination times, maternal education status, maternal marital status, maternal age, and parity were used as covariates.

### Fertility policy adjustment

On the 28^th^ of December 2013, the Chinese government announced that a couple was allowed to have a second child if either parent was an only child. In January 2016, in order to alleviate the decreasing birth rate, the government implemented a “universal two-child policy”, allowing each couple to have a second child [21]. Based on China’s fertility policy change, this study was categorised into three periods: before 2014, 2014–2015, and 2016–2020.

### Statistical analysis

We calculated the number and rate of stillbirths, SGA, and low Apgar scores according to maternal sociodemographic and obstetric characteristics. Because the large urban hospitals were overrepresented in the NMNMSS, we weighted the rate of each adverse outcome based on the sampling distribution of the population according to the 2010 census of China, which has been described previously [17]. Demographical, maternal, and infant characteristics were summarised and compared according to different adverse perinatal outcomes. The associations between each adverse perinatal outcome and maternal sociodemographic and obstetric characteristics were analysed by logistic regression, considering the cluster effect at the hospital and individual level.

Interrupted time series analysis (ITSA) was used to compare the differences before and after the fertility policy change. The ITSA model compares the intervention’s effect in each phase while taking autocorrelation into consideration [22]. In this study, the three phases were analysed. The ITSA model assumes the following equations:

$${Y}_{t}={\beta }_{0}+{\beta }_{1}\times {T}_{t}+{\beta }_{2}\times {X}_{t1}+{\beta }_{3}\times {T}_{t}{X}_{t1}+{\beta }_{4}\times {X}_{t2}+{\beta }_{5}\times {T}_{t}{X}_{t2}+\sum {\beta }_{j}Covariates+{\varepsilon }_{t}$$

In this equation, ${Y}_{t}$ was the outcome of twin birth, stillbirth, SGA, and low Apgar score rates among twins. ${\beta }_{0}$ represented the initial level of each outcome. ${\beta }_{1}$ was the change in each outcome before the start of the intervention. ${\beta }_{2}$ and ${\beta }_{4 }$ were the levels of each outcome in the period immediately after the intervention was implemented in 2014 and 2016 (immediate effect), respectively. ${\beta }_{3}$ was the difference in each outcome between 2012–2013 and 2014–2015. ${\beta }_{5}$ was the difference in each outcome between 2014–2015 and 2016–2017 (long term effect). In the ITSA analysis, to exclude the confounding factors of gestational age, we used the time for women to become pregnant (Time for women to become pregnant = Time for women delivered-gestational week*7-gestational day).

To demonstrate the trend of each perinatal outcome by gestational age over the years, graphs indicating the proportion of infants against each outcome by gestational age were created. Each graph included a local regression curve that was fit using data proportions and a smoothing parameter of 0.4 for the assessment of trends [23]. To analyse the trend of each outcome according to gestational age, a multivariate generalised linear model (GLM) was used.

To analyse the risk of stillbirth, SGA, and low Apgar scores at differing gestational ages, two different reference methods were used. One reference was calculated using the rate of each perinatal outcome at 37 weeks of gestational age; the other method to calculate the reference rate at a given gestational age was to use the number of infants born at that gestational age or more (women who go on to deliver after a longer gestation period by any mode of onset) as the denominator [24, 25]. The risk of each perinatal outcome was analysed using the multivariate GLM, considering the cluster effect at the hospital and individual level. Models were adjusted for covariates such as area classification, geographic location, hospital level, infant birth year, maternal age, education, marriage, parity, prenatal examination and complicating conditions, twin birth sequence, and weight imbalance.

The summaries and analysis work were completed using SAS statistical software (version 9.4; SAS Institute) and Stata version 15.1 (Stata Corp., TX, USA). A two-sided *P* value of less than 0.05 was considered significant.

## Results

**Fig. 1:**
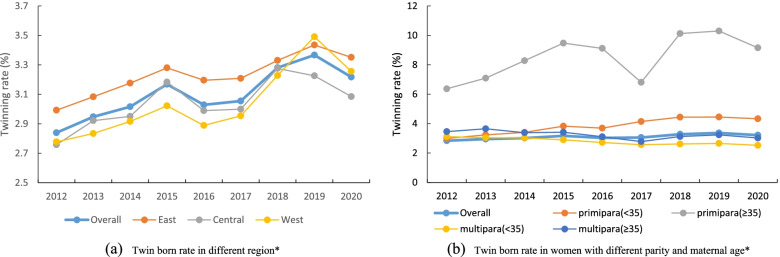
Twin born rate in China. **A** Twin born rate in different region*. **B** Twin born rate in women with different parity and maternal age*. *Rates are n (%). All results were adjusted for the sampling distribution of the population

The twinning rate was 3.53% between 2012 and 2020, and after weighting for the population sample distribution, the adjusted twinning rate was 3.10%. The twinning rate increased from 2.84% in 2012 to 3.22% in 2020. The twinning rate increased most at gestational ages between 28 and 31 weeks by 2.66% (95% CI 2.02% to 3.30%) (Table 1). Between 2016 and 2020, the West region increased most out of all of the regions. The change in twinning rate among primipara and multipara decreased over the same period. The twinning rate of primiparas with advanced maternal age was higher than that at the national level (Fig. 1). Little difference in the change of twinning rate change was observed during the separate fertility policy periods (Table 2).

**Table 1:** Twinning rate, Stillbirth, SGA and low apgar score rate (%) and its annual change in China, 2012–2020

	Year ^a^									Annual rate change (95% CI) ^b^
	2012	2013	2014	2015	2016	2017	2018	2019	2020	
Twin born rate										
overall	2.84	2.95	3.02	3.17	3.03	3.05	3.28	3.37	3.22	1.75%[1.57%,1.93%]
28-31w	15.19	15.70	17.89	17.48	17.68	17.35	18.30	19.73	18.91	2.66%[2.02%,3.30%]
32-33w	20.21	20.68	22.24	22.16	22.62	22.34	23.59	24.84	23.52	2.17%[1.64%,2.71%]
34-36w	19.33	20.46	21.26	21.81	21.07	22.08	23.49	23.82	22.63	2.19%[1.95%,2.44%]
37-38w	4.30	4.60	4.71	4.99	4.46	4.46	4.76	4.97	4.24	0.19%[-0.08%,0.46%]
≥ 39w	0.50	0.47	0.42	0.40	0.32	0.29	0.24	0.20	0.14	-13.11%[-13.76%,-12.46%]
Stillbirth										
overall	2.20	2.30	2.10	1.86	1.71	1.53	1.44	1.29	1.40	-7.35%[-8.35%,-6.24%]
28-31w	12.16	12.85	10.55	10.48	9.68	7.05	8.46	7.18	7.82	-7.16%[-9.05%,-5.24%]
32-33w	4.41	4.79	4.32	3.71	3.79	3.61	3.45	2.69	3.40	-5.38%[-7.86%,-2.83%]
34-36w	1.74	1.94	1.81	1.39	1.23	1.32	1.00	0.92	0.90	-9.72%[-11.41%,-8.00%]
37-38w	1.03	0.98	0.92	0.84	0.71	0.66	0.55	0.55	0.55	-9.05%[-11.27%,-6.78%]
≥ 39w	1.96	1.71	1.55	1.98	1.81	1.81	2.14	1.89	3.00	2.97%[-1.31%,7.43%]
SGA										
overall	8.91	8.74	8.09	7.97	7.85	7.29	7.11	6.81	6.69	-3.67%[-4.14%,-3.21%]
28-31w	8.85	8.99	8.78	9.10	8.92	7.41	8.25	7.86	9.32	-0.92%[-2.78%,0.99%]
32-33w	9.70	10.55	10.04	10.06	10.37	9.21	10.24	9.83	8.82	-0.99%[-2.42%,0.45%]
34-36w	9.39	9.23	8.50	8.49	8.32	7.47	7.14	7.35	6.84	-3.96%[-4.67%,-3.25%]
37-38w	7.98	7.68	7.31	6.87	6.53	6.39	6.22	5.47	5.59	-4.64%[-5.42%,-3.85%]
≥ 39w	10.37	10.02	8.24	9.14	10.33	10.28	9.24	9.34	10.79	-0.02%[-2.00%,1.99%]
Low apgar score (< 4)										
overall	1.09	1.01	0.94	0.85	0.87	0.76	0.58	0.55	0.54	-8.70%[-10.18%,-7.18%]
28-31w	7.70	9.11	7.50	6.96	6.36	6.26	6.12	4.26	4.91	-7.56%[-10.03%,-5.02%]
32-33w	2.40	1.82	2.04	2.02	2.24	2.13	1.42	1.74	1.05	-5.39%[-8.95%,-1.68%]
34-36w	0.85	0.67	0.60	0.53	0.53	0.51	0.25	0.30	0.32	-11.71%[-15.06%,-8.22%]
37-38w	0.45	0.34	0.31	0.23	0.28	0.13	0.11	0.09	0.11	-17.61%[-21.73%,-13.26%]
≥ 39w	0.61	0.61	0.50	0.70	0.74	0.61	0.35	0.50	0.27	-2.84%[-10.35%,5.31%]

^a^ the number of stillbirth per 100 deliveries or the number of SGA/Low apgar score (< 4) infants per 100 live born. All results were adjusted for the sampling distribution of the population. ^b^ Adjusted for the clustering of births within hospitals

**Table 2:** Analysis of fertility policy in different period

	Time point^a^			
	2014.1		2016.1	
	immediate effect	long term effect	immediate effect	long term effect
Twin born rate	0.00[-0.52,0.53]	-0.05[-0.11,0.01]	-0.48[-2.96,2.00]	-0.03[-0.12,0.07]
Adverse perinatal outcome				
Stillbirth	-0.27[-0.84,0.31]	0.02[-0.04,0.07]	-0.17[-0.76,0.42]	0.01[-0.02,0.04]
SGA	-0.12[-0.78,0.54]	0.03[-0.02,0.08]	-0.13[-0.83,0.57]	-0.02[-0.05,0.02]
Low apgar score (< 4)	-0.40[-1.19,0.39]	0.02[-0.03,0.08]	-0.56[-1.28,0.16]	0.01[-0.03,0.06]

All results were adjusted for the sampling distribution of the population. Adjusted for hospital level, area classification, advanced maternal age (≥ 35), maternal education, parity and prenatal examination as covariates

^a^ time in this analysis was the time that women got pregnant

The stillbirth, SGA, and low Apgar score rates among twins were 1.84%, 7.77%, and 0.80%, respectively. After weighting for population distribution, the adjusted rates were 1.75%, 7.70%, and 0.79%, respectively. From 2012 to 2020, all three parameters showed a decreasing trend. The adjusted stillbirth rate decreased from 2.22% in 2012 to 1.40% in 2020, with an annual rate of change of -7.35% (95% CI -8.35% to -6.24%). The adjusted SGA rate decreased from 8.91% in 2012 to 6.69% in 2020, with an annual rate of change of -3.67% (95% CI -4.14% to -3.21%). The adjusted low Apgar score rate decreased from 1.09% in 2012 to 0.54% in 2020, with an annual rate of change of -8.70% (95% CI -10.18% to -7.18%) (Table 1). Little differences in changes of stillbirth, SGA, and low Apgar scores rates were observed in the separate fertility policy periods (Table 2).

There were 221,293 pregnant women with 442,268 infants enrolled in this study (318 infants were excluded for an unknown status of birth (live birth or stillbirth)). Most of the enrolled pregnant women were primiparas (62.81%) aged 20–34 years (79.70%) from urban areas (78.03%). The majority of them had at least five prenatal examinations (84.17%) and were educated at a tertiary level or above (94.53%) (Table 3).

**Table 3:** Baseline demographics of the population of 442,268 twins born in China

	Number of pregnancies	Stillbirth		SGA		Low Apgar score (< 4)	
		Rate (per 100 deliveries)	Adjust odds ratio(95% CI)^ab^	Rate (per 100 live births)	Adjust odds ratio(95% CI) ^ab^	Rate (per 100 live births)	Adjust odds ratio(95% CI) ^ab^
N(%)	442,268(100.00%)	8145(1.75%)	-	31,408(7.70%)	-	3346(0.79%)	-
Area classification							
urban	345,102(78.03%)	6686(1.89%)	ref	25,833(7.54%)	ref	2601(0.77%)	ref
rural	97,166(21.97%)	1459(1.51%)	0.97[0.88,1.08]	7575(7.99%)	1.05[1.01,1.10]	765(0.81%)	1.53[1.33,1.76]
Geographic location							
east	123,856(28.00%)	1862(1.49%)	0.81[0.74,0.88]	7879(6.61%)	0.94[0.90,0.97]	823(0.68%)	0.89[0.79,1.00]
central	172,905(39.10%)	3158(1.74%)	ref	12,634(7.34%)	ref	1249(0.73%)	ref
west	145,507(32.90%)	3125(2.11%)	1.17[1.08,1.27]	12,895(9.60%)	1.36[1.32,1.41]	1294(0.99%)	1.38[1.24,1.53]
Hospital level							
level 1	30,580(6.91%)	467(1.43%)	0.86[0.75,1.00]	2473(8.30%)	0.91[0.86,0.96]	190(0.69%)	0.76[0.62,0.92]
level 2	123,667(27.96%)	1746(1.47%)	0.95[0.87,1.05]	9043(7.66%)	0.91[0.88,0.95]	853(0.74%)	0.86[0.76,0.99]
level 3	288,021(65.12%)	5932(1.99%)	ref	21,892(7.65%)	ref	2323(0.82%)	ref
Year of delivery							
2012–2013	119,646(27.05%)	2727(2.16%)	ref	10,019(8.61%)	ref	1130(0.96%)	ref
2014–2015	101,860(23.03%)	2008(1.86%)	0.85[0.78,0.93]	7913(7.96%)	0.98[0.94,1.02]	859(0.85%)	0.86[0.76,0.97]
2016–2017	109,305(24.71%)	1724(1.50%)	0.68[0.61,0.74]	7872(7.33%)	0.95[0.91,0.99]	737(0.71%)	0.77[0.68,0.87]
2018–2020	111,457(25.20%)	1686(1.43%)	0.60[0.54,0.66]	7604(6.78%)	0.92[0.88,0.95]	640(0.58%)	0.62[0.54,0.70]
Maternal age							
< 20	4506(1.02%)	182(3.66%)	1.50[1.19,1.89]	692(15.81%)	1.81[1.61,2.04]	82(1.73%)	1.51[1.08,2.11]
20–25	47,481(10.74%)	1308(2.48%)	1.30[1.18,1.44]	4993(10.64%)	1.34[1.28,1.40]	535(1.14%)	1.28[1.12,1.47]
25–30	151,693(34.30%)	2797(1.72%)	ref	11,478(7.69%)	ref	1096(0.73%)	ref
30–35	153,274(34.66%)	2264(1.41%)	0.82[0.75,0.89]	10,012(6.51%)	0.84[0.82,0.87]	935(0.63%)	0.91[0.81,1.02]
≥ 35	72,852(16.47%)	1278(1.70%)	0.84[0.76,0.94]	5069(7.14%)	0.90[0.86,0.94]	549(0.77%)	0.95[0.82,1.09]
unknown	12,462(2.82%)	316(2.56%)	-	1164(9.67%)	-	169(1.47%)	-
Maternal marriage							
Single/widower/divorced/cohabitation	4686(1.06%)	154(2.99%)	1.11[0.84,1.45]	511(11.44%)	1.14[0.99,1.30]	62(1.25%)	0.95[0.66,1.39]
married	437,464(98.91%)	7990(1.74%)	ref	32,886(7.66%)	ref	3304(0.78%)	ref
unknown	118(0.03%)	1(1.08%)	-	11(10.32%)	-		-
Maternal education							
college or above	193,505(43.75%)	2925(1.45%)	0.85[0.79,0.92]	12,884(6.64%)	0.86[0.84,0.89]	1056(0.55%)	0.80[0.72,0.89]
secondary school	223,568(50.55%)	4509(1.86%)	ref	18,207(8.22%)	ref	1979(0.88%)	ref
primary school or below	13,704(3.10%)	434(3.00%)	1.30[1.12,1.53]	1557(11.78%)	1.40[1.30,1.51]	234(1.75%)	1.50[1.24,1.82]
unknown	11,491(2.60%)	277(2.36%)	-	760(7.18%)	-	97(0.96%)	-
Parity							
multipara	163,110(36.88%)	3444(1.96%)	1.34[1.24,1.44]	11,382(7.12%)	0.86[0.83,0.89]	1425(0.90%)	1.30[1.18,1.43]
primipara	277,807(62.81%)	4642(1.60%)	ref	22,017(8.10%)	ref	1937(0.70%)	ref
unknown	1351(0.31%)	59(4.32%)	-	9(5.85%)	-	4(2.62%)	-
Inadequate prenatal examination							
< 5 times	45,884(10.37%)	1691(3.40%)	1.50[1.38,1.64]	4562(10.28%)	1.27[1.22,1.33]	842(1.83%)	1.56[1.40,1.75]
≥ 5 times	372,236(84.17%)	5806(1.48%)	ref	27,131(7.36%)	ref	2306(0.63%)	ref
unknown	24,148(5.46%)	648(2.63%)	-	1715(7.58%)	-	218(1.01%)	-
Maternal complications							
no	171,954(38.88%)	2806(1.56%)	ref	11,895(7.12%)	ref	1048(0.63%)	ref
medical complication	77,773(17.59%)	1142(1.42%)	0.90[0.81,1.00]	5430(7.07%)	0.97[0.93,1.01]	651(0.87%)	1.37[1.20,1.57]
antepartum complication	192,541(43.53%)	4197(2.05%)	1.07[0.99,1.15]	16,083(8.48%)	1.09[1.06,1.13]	1667(0.89%)	1.20[1.08,1.32]
Gestational week							
≤ 31w	26,806(6.06%)	2520(9.48%)	12.13[10.87,13.54]	2048(8.58%)	0.98[0.92,1.04]	1444(6.47%)	29.25[25.14,34.03]
32-33w	35,338(7.99%)	1324(3.78%)	4.45[3.95,5.01]	3361(9.88%)	1.18[1.12,1.24]	616(1.90%)	7.86[6.65,9.28]
34-36w	173,638(39.26%)	2356(1.34%)	1.81[1.64,1.99]	13,704(8.02%)	1.13[1.10,1.17]	798(0.49%)	2.21[1.90,2.57]
37-38w	181,673(41.08%)	1398(0.75%)	ref	11,784(6.65%)	ref	359(0.22%)	ref
≥ 39w	24,813(5.61%)	547(1.87%)	1.69[1.45,1.97]	2511(9.68%)	1.29[1.21,1.38]	149(0.59%)	1.81[1.42,2.31]
Infants weight discordance							
no	360,526(81.52%)	2809(0.78%)	ref	15,492(4.37%)	ref	2237(0.63%)	ref
yes	74,604(16.87%)	3587(4.50%)	5.11[4.77,5.48]	17,772(24.95%)	7.32[7.13,7.53]	1077(1.49%)	1.90[1.73,2.10]
unknown	7138(1.61%)	1749(24.58%)	-	144(6.64%)	-	52(2.49%)	-

Data are n (%) unless otherwise stated. All results were adjusted for the sampling distribution of the population

^a^Adjusted for the clustering of births within hospitals and pregnant woman individuals

^b^Factors listed in Table1 were used as covariates for adjustment

**Fig. 2:**
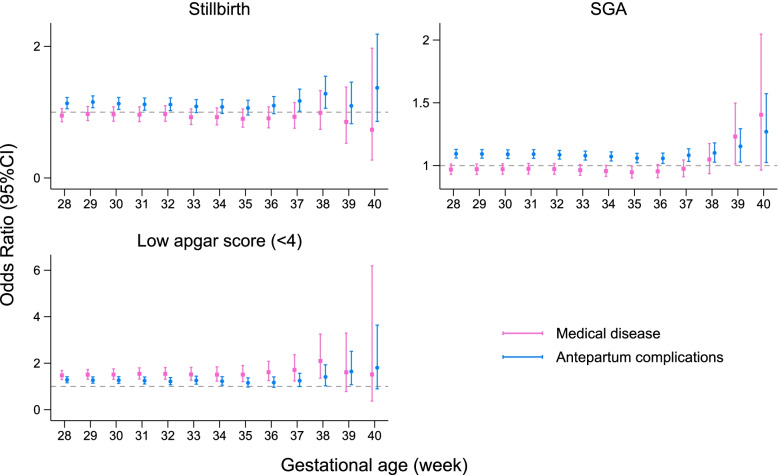
Association between maternal complications and adverse perinatal outcomes in in different gestational age*. * Foetuses staying in utero were used as reference in separate gestational ages, women in medical diseases group or antepartum complications group were compared with uncomplicated women. All results were adjusted for the sampling distribution of the population and clustered of births within hospitals and pregnant woman individuals. Covariates were adjusted as area classification, geographic location, hospital level, infants birth year, maternal age, education, marriage, parity, prenatal examination, twins born sequence and weight imbalance

Compared with infants remaining in utero, the birth of twins at any time between 28 and 35 weeks of gestation was associated with an increased risk of stillbirth (odds ratio [OR] 10.18, 95% confidence interval [CI] 8.67–11.96 at 28 weeks; OR 1.61, 95% CI 1.43–1.82 at 35 weeks). Conversely, the birth of twins at any time between 37 and 39 weeks of gestation was associated with a decreased risk of stillbirth (OR 0.62, 95% CI 0.53–0.71 at 37 weeks; OR 0.67, 95% CI 0.50–0.90 at 39 weeks) (Table 4). In separate maternal complication subgroups, infants born at any gestational week between 37 and 39 weeks were associated with a decreased risk of stillbirth among mothers with antepartum complications. Infants born at 37 weeks of gestation were associated with a decreased risk of stillbirth among mothers with medical diseases (STable 1). At gestational ages between 28 and 30 weeks, infants with mothers from the antepartum complications group were associated with an increased risk of stillbirth compared with uncomplicated women (Fig. 2). When compared with infants born at 37 weeks, any gestational age from 28 to 41 weeks (except 37 weeks) was associated with an increased risk of stillbirth. The risk of stillbirth increased most at 28 weeks (OR 26.28, 95% CI 21.89–31.54) and increased the least at 38 weeks (OR 1.26, 95% CI 1.08–1.48]) (Table 4).

**Table 4:** Risk of stillbirth, SGA and Low apgar score (< 4) in different gestational age

	Stillbirth				SGA				Low apgar score (< 4)			
Gestational week	No. With Outcome/Total No. in Group (%)		Compared with infants born at 37 weeks Adjusted OR	Compared with fetus remaining in utero Adjusted OR	No. With Outcome/Total No. in Group (%)		Compared with infants born at 37 weeks Adjusted OR	Compared with fetus remaining in utero Adjusted OR	No. With Outcome/Total No. in Group (%)		Compared with infants born at 37 weeks Adjusted OR	Compared with fetus remaining in utero Adjusted OR
	Ongoing Pregnancies	Delivered			Ongoing Pregnancies	Delivered			Ongoing Pregnancies	Delivered		
28	7494/437984 (1.63)	651/4284 (15.27)	26.28 [21.89,31.54]	10.18 [8.67,11.96]	33,162/430490 (7.65)	246/3633 (6.74)	0.81 [0.67,0.96]	0.72 [0.61,0.86]	3023/430490 (0.70)	423/3633 (12.46)	65.60 [52.76,81.57]	18.75 [15.92,22.09]
29	6878/432714 (1.52)	616/5270 (11.67)	17.12 [14.36,20.41]	7.14 [6.12,8.32]	32,828/425836 (7.65)	334/4654 (7.09)	0.88 [0.76,1.01]	0.78 [0.68,0.90]	2631/425836 (0.62)	392/4654 (8.72)	40.68 [32.74,50.54]	13.23 [11.23,15.59]
30	6240/425331 (1.41)	638/7383 (8.74)	11.71 [9.81,13.97]	5.36 [4.60,6.25]	32,180/419091 (7.62)	648/6745 (9.52)	1.09 [0.98,1.21]	0.98 [0.88,1.08]	2281/419091 (0.55)	350/6745 (5.45)	24.96 [20.14,30.92]	9.37 [7.98,11.00]
31	5625/415462 (1.30)	615/9869 (6.33)	8.44 [7.13,9.98]	4.25 [3.68,4.91]	31,360/409837 (7.60)	820/9254 (8.86)	1.05 [0.96,1.15]	0.94 [0.86,1.02]	1975/409837 (0.49)	306/9254 (3.58)	15.79 [12.63,19.75]	6.89 [5.79,8.20]
32	4946/401036 (1.19)	679/14426 (4.70)	5.90 [5.02,6.92]	3.26 [2.85,3.73]	29,951/396090 (7.52)	1409/13747 (10.13)	1.23 [1.14,1.33]	1.10 [1.03,1.19]	1663/396090 (0.43)	312/13747 (2.38)	9.90 [7.95,12.33]	4.95 [4.18,5.86]
33	4301/380124 (1.09)	645/20912 (3.15)	4.21 [3.60,4.92]	2.57 [2.25,2.93]	27,999/375823 (7.43)	1952/20267 (9.43)	1.19 [1.12,1.27]	1.08 [1.01,1.14]	1339/375823 (0.37)	324/20267 (1.59)	6.53 [5.26,8.10]	3.85 [3.26,4.55]
34	3520/345629 (0.99)	781/34495 (2.26)	3.12 [2.69,3.62]	2.13 [1.88,2.41]	24,830/342109 (7.26)	3169/33714 (9.26)	1.25 [1.18,1.32]	1.15 [1.09,1.21]	1038/342109 (0.32)	301/33714 (0.95)	4.36 [3.52,5.39]	3.15 [2.66,3.73]
35	2769/294590 (0.91)	751/51039 (1.46)	2.14 [1.86,2.46]	1.61 [1.43,1.82]	20,638/291821 (7.10)	4192/50288 (8.29)	1.18 [1.12,1.23]	1.10 [1.05,1.15]	785/291821 (0.29)	253/50288 (0.54)	2.27 [1.82,2.84]	1.87 [1.55,2.25]
36	1945/206486 (0.91)	824/88104 (0.93)	1.42 [1.23,1.63]	1.12 [0.99,1.26]	14,295/204541 (7.03)	6343/87280 (7.27)	1.11 [1.06,1.16]	1.06 [1.02,1.10]	522/204541 (0.28)	263/87280 (0.30)	1.35 [1.09,1.68]	1.19 [0.99,1.43]
37	1119/77065 (1.29)	826/129421(0.64)	ref	0.62 [0.53,0.71]	6273/75946 (8.08)	8022/128595 (6.31)	ref	0.86 [0.82,0.91]	284/75946 (0.38)	238/128595(0.21)	ref	0.83 [0.65,1.06]
38	547/24813 (1.87)	572/52252 (0.99)	1.26 [1.08,1.48]	0.59 [0.49,0.71]	2511/24266 (9.59)	3762/51680 (7.29)	1.06 [1.01,1.12]	0.76 [0.71,0.82]	154/24266 (0.61)	130/51680 (0.26)	0.97 [0.75,1.26]	0.51 [0.38,0.69]
39	197/6899 (2.41)	350/17914 (1.66)	1.68 [1.38,2.04]	0.67 [0.50,0.90]	742/6702 (10.18)	1769/17564 (9.35)	1.33 [1.23,1.43]	0.91 [0.81,1.03]	57/6702 (0.87)	97/17564 (0.50)	1.49 [1.10,2.02]	0.55 [0.36,0.84]
40	41/1093 (3.37)	156/5806 (2.24)	2.05 [1.56,2.68]	0.61 [0.36,1.04]	121/1052 (10.13)	621/5650 (10.19)	1.35 [1.20,1.52]	1.07 [0.80,1.41]	16/1052 (1.34)	41/5650 (0.79)	2.27 [1.49,3.46]	0.65 [0.30,1.39]
41		41/1093 (3.37)	3.26 [2.10,5.07]			121/1052 (10.13)	1.28 [0.97,1.68]			16/1052 (1.34)	3.30 [1.72,6.32]	

All results were adjusted for the sampling distribution of the population and clustered of births within hospitals and pregnant woman individuals. Covariates were adjusted as area classification, geographic location, hospital level, infants birth year, maternal age, education, marriage, parity, prenatal examination and maternal health condition, twins born sequence and weight imbalance

Twins born at 37 and 38 weeks of gestational age were associated with a decreased risk of SGA when compared with ongoing pregnancies (OR 0.86, 95% CI 0.82–0.91 at 37 weeks; OR 0.76, 95% CI 0.71–0.82 at 38 weeks), while infants born any time between 32 to 36 weeks of gestational age were associated with an increased risk of SGA (OR 1.23, 95% CI 1.14–1.33 at 32 weeks; OR 1.11, 95% CI 1.06–1.16 at 36 weeks) (Table 4). Compared with ongoing pregnancies, there was a decreased risk of SGA in infants born at gestational ages between 37 and 38 weeks with mothers from the antepartum complications group, as well as in infants born at gestational ages between 28 and 29 weeks, and 37 and 38 weeks with mothers from the medical disease group (STable 2).

Infants born at any time between 28 and 35 weeks were associated with an increased risk of low Apgar scores, while infants born at any time between 38 to 39 weeks were associated with a decreased risk of low Apgar scores compared with ongoing pregnancies (OR 0.51, 95% CI 0.38–0.69 at 38 weeks; OR 0.55, 95% CI 0.36–0.84 at 39 weeks) (Table 4). In separate maternal complication subgroups, infants born at 38 or 39 weeks of gestation were associated with a decreased risk of low Apgar scores among mothers with antepartum complications (STable 3). However, when compared with infants born at 37 weeks of gestational age, infants born in any week between 28 to 41 weeks (excluding 38 weeks of gestational age) were associated with an increased risk of low Apgar scores (Table 4).

## Discussion

In this study, the twinning rate, rate of stillbirth, SGA, and low Apgar score among Chinese twins were demonstrated at different gestational ages between 2012 and 2020. Twin infants delivered at 37 weeks of gestational age had a decreased risk of stillbirth, SGA, and low Apgar scores. However, women with medical diseases or antepartum complications should be cautious, due to an increased risk of adverse outcomes. To the best of our knowledge, this is the first in-depth study demonstrating the trends and risks of stillbirth, SGA, and low Apgar scores among twins in China after the fertility policy change.

The Chinese twin birth rate in our study was close to the rate in the United States (US, 32.6‰) and higher than that in the UK (15 twins birth for every 1000 pregnant women) in 2018 [26]. One study showed that twins born in Asia accounted for 42% of all twins in the world from 2010 to 2015; however, the twinning rate in Asia was 9.2‰, which is lower than that in other global regions [27]. In that study, the Chinese twinning rate was based on Deng’s research, which reports a lower rate than in our results [3]. This difference between the twinning rates may be caused by different methods of data collection. In Deng’s study, the twinning rate was estimated using aggregative data, where there is a high chance that underreporting occurred; whilst in our study, the twinning rate was calculated using individual data, and the number of foetuses was recorded per individual woman. On the other hand, the higher rates in our study may be driven by the NMNMSS oversampling large referral hospitals in urban districts, where twin birth rates and stillbirth rates were higher than in smaller hospitals. In this study, we weighted the rate of each adverse perinatal outcome by the population distribution in urban and rural areas. The twinning and stillbirth rates before the adjustment of the sample distribution were 3.52% and 1.84%. This method for weighting by sampling distribution of the population was also mentioned in a previous study that demonstrated stillbirth and preterm birth rates in China [17, 20].

The increasing twinning rate was also observed in Deng’s research [3]. However, little difference in the changes in twinning rate was observed in the different fertility policy periods. This differed from the overall number of new-borns. Previous research has shown that the change in China’s fertility policy promoted 5.4 million new-borns during the “universal two-child policy” [4]. Multipara contributed to the increased birth-rate in that study, while in our research, we found the inverse regarding the twinning rate among primipara and multipara. The twinning rate increased in primiparas from 2012 to 2020. However, the twinning rate among multiparas decreased slightly during the same time. It is well known that an increase in twinning birth is closely related to the development of assisted reproductive technology (ART). The higher rate of twinning among primiparas with advanced maternal age may be related to the development of ART in China [28]. This was in accordance with previous research demonstrating that increased maternal age, ART, and an increased number of females who completed a college or higher degree may be responsible for the increase in the rates of multiple births [29, 30]. Thus, we may conclude that fertility policy change has had little effect on the increasing twinning rate. However, since a higher rate of conceived twins was observed among the primiparas with advanced maternal age in this study, we cannot ignore the potential risk of a twin pregnancy. In this study, we found a decreased risk of adverse perinatal outcomes among women with advanced age. This was contrary to the common belief that advanced age is an independent risk factor for adverse perinatal outcomes [31]. The lower risk of perinatal outcomes in our results may be attributed to the decreased fertility in older adult women and predominantly getting pregnant with the aid of ART, subsequently resulting in a higher probability of twin pregnancy. Successful embryo implantation and live feotuses are of high priority, potentially resulting in better adherence to medical advice, increased focus on prenatal examinations, nutritional assessments and self-monitoring of maternal and fetal health. An increased risk of maternal complications in women may result in increased cautiousness during pregnancy. Prompt hospital visits were likely made at any sign of discomfort or abnormalities for the mothers or the feotuses. These measures can greatly decrease the risk of stillbirth and SGA. In our study, over 80% of the women went through at least five times of prenatal examinations during their pregnancies. Our results were also consistent with another research that indicated a decreased risk of infant death in twin pregnancy in older women [32]. Our results may serve as a caution to pregnant women, especially those undergoing ART. We strongly suggest that a systemic physical examination be conducted and stringent assessment of the probability of success and the safety of ART be carried out before the process is initiated. Secondly, with the development of ART and greater chances of successful implantation, a single embryo transfer may be more suitable for women of advanced age. Besides, regular and rigorous prenatal examinations during the perinatal period are also encouraged. Nutritional assessment and self-monitoring may also have a role in reducing adverse perinatal outcomes in women of advanced age [33].

The adjusted stillbirth rate of twins was 1.75%, which was higher than that reported by Deng, but lower than that of the Zhejiang province [3, 34]. Additionally, it was lower than in developing countries, such as Zambia (39.4‰ between 2006–2013) [35] and higher than in developed countries such as the US (15.8‰, 2005–2006) [36] and the UK (6.16‰ in 2016) [37]. From 2012 to 2020, the stillbirth, SGA, and low Apgar score rates showed decreasing trends. However, the fertility policy has been observed to have little effect on the adverse perinatal outcomes of twins. These parameters may have benefited from China’s social and economic development, and government strategies for strengthening pregnancy health management and improving maternal and neonatal outcomes, such as maternal and foetal physical examinations and health evaluations; referral of women having a high risk pregnancy, equal access to basic public health services, the establishment of near miss new-born care centres, and guidance for the clinical management of twin pregnancies [38, 39]. China’s government made significant efforts to improve maternal and perinatal outcomes by promoting perinatal examinations. The National Basic Public Health Service Project has offered rural pregnant women five times of the perinatal examination appointments for free [40]. Our results showed that 84.17% of the twin infants underwent at least five times prenatal examinations during pregnancies. Infants born with inadequate prenatal examinations (i.e., less than five) had higher risk of stillbirth, SGA, and low Apgar scores. Besides, the National Health Commission of China has promoted health education by popularizing basic health knowledge applicable to women pre-marriage, pre-pregnancy and during pregnancy using several mediums, thereby increasing the knowledge and awareness of health care for women and families. The National Health Commission of China has also organized the “Neonatal Asphyxia Resuscitation Training Program” to reduce the mortality and disability rates of neonatal asphyxia since 2004. The training was first conducted in the “Reducing Maternal Mortality and Eliminating Neonatal Tetanus” program province, and was subsequently expanded to other provinces by offering provincial teacher training, teaching materials, and technical support [41]. Now, the “Neonatal Asphyxia Resuscitation Techniques” have been adopted as a basic skill test for midwives and doctors. Additionally, standardized training of obstetricians during their first three years of practice in China has enhanced the standardization of obstetric practices and the improvement of overall obstetrics technology and service quality.

While the stillbirth, SGA, and low Apgar score rates were on a decreasing trend over the years, the annual decreasing rate for various gestational ages was different, decreasing the most between 37 and 38 weeks. A previous multiple-population based study indicated that births delivered between 37 and 39 weeks had the lowest risk of perinatal mortality and morbidity [9]. However, the “optimal gestational age” rarely considered maternal and perinatal complications [11, 12]. In this study, we classified enrolled women as having uncomplicated pregnancies (including those with maternal health conditions), pregnancies with medical diseases, and pregnancies with antepartum complications. To select the optimum gestational age with the lowest risk of stillbirth, SGA, and a low Apgar score, we used two methods to estimate the risk of each adverse outcome. One was comparing foetuses delivered at different gestational weeks with foetuses delivered at 37 gestational weeks (the conventional method); the other was comparing foetuses delivered at any gestational week with ongoing pregnancies. The latter method was used as a measurement of short-term risk [42]. Using this method of risk estimation, infants born at any time between 37 and 39 gestational weeks were associated with a decreased risk of stillbirth. And in the conventional method, infants born at 37 weeks of gestational age were associated with the lowest risk of stillbirth. Our results for stillbirth were generally consistent with those of previous studies [8, 42]. Our results showed that infants born at around 37 weeks of gestation had the lowest risk of stillbirth, while infants born at shorter or longer gestational ages, especially those born at extremely short gestational ages had higher risks of stillbirth. This is possibly due to twin pregnancies being associated with increased risks of stillbirth and preterm labour. Monochorionic pregnancies negatively affect the in-utero survival of twins, even in monochorionic-diamniotic twins without abnormalities [43]. Monochorionic twins also experience complications like twin to twin transfusion syndrome and selective intrauterine growth retardation. These complications increase the risk of stillbirths. Premature infants have higher risk of stillbirth [44]. Twin pregnancies have higher risk of preterm labour than singleton pregnancies, to the extent that over half of the women pregnant with twins give birth before 37 weeks of gestation [7]. Furthermore, twin pregnancies are generally more likely when using ART [45]. The women who underwent ART were either of advanced age or had declining fertility. It is well-established that advanced age is an independent risk factor for adverse maternal and perinatal outcomes [31]. Women of advanced ages had higher risk of stillbirth. In our results, women with antepartum complications had increased risks of stillbirth when the gestational age was less than 33 weeks. We assumed that these could possibly be a form of natural selection of the implanted embryos to alleviate the additional burden of not successfully sustaining twin pregnancies in women with antepartum complications, leading to stillbirths at an early gestational age. Research has showed that ART-conceived pregnancies were associated with higher risk of birth defects and stillbirths [45, 46]. One study found that in comparison with the gestational ages of stillborn infants of women who conceived spontaneously, the mean gestational age at stillbirth was lower for infants conceived through fertility treatments, suggesting different aetiologies of stillbirth [47]. In addition, we found that live-born twins delivered at a gestational age between 37 and 38 weeks were associated with a lower risk of SGA and low Apgar scores. Furthermore, we found that in reference to uncomplicated pregnancies, women with antepartum complications were associated with an increased risk of giving birth to SGA infants in different gestational weeks compared with ongoing pregnancies. Live-born infants born at any time between 28 and 39 weeks among women with medical diseases, and 28 and 34 weeks among women with antepartum complications were associated with an increased risk of low Apgar scores. Although the increased risk of SGA or low Apgar scores were in accordance with previous research, few of them analysed these associations at different gestational ages [48–51]. Besides, in subgroup analysis, infants born at 37 or 38 weeks of gestational age were associated with a decreased risk of stillbirth, SGA, and low Apgar score when compared with ongoing pregnancies. Thus suggesting that 37 and 38 weeks were the optimal gestational ages for decreased risk of stillbirth, SGA, and low Apgar scores when compared with ongoing pregnancies.

The strength of this study was in demonstrating the twinning rate and health condition of twins in an adequate sample size from 2012 to 2020 and analysing the health conditions of twins at different gestational ages. However, there are still limitations to this study. Firstly, the weighting method of population distribution may not have been fully adjusted for the oversampling of high-level hospitals when estimating the stillbirth, SGA, and low Apgar rate of twins. Secondly, we could not distinguish whether the twins were monochorionic or dichorionic, and the type of chorionicity was an important factor for stillbirth and perinatal health in twin born infants. Further studies could explore different types of twins.

## Conclusions

The twinning rate in China showed an increasing trend from 2012 to 2020, and the adverse perinatal outcomes of twins showed a decreasing trend during the same period. Changes in the fertility policy have little effect on the twinning rate or the rate of adverse perinatal outcomes such as stillbirth, SGA, or rate of low Apgar scores. The optimal gestational age for twins to lower the risk of stillbirth, SGA, and low Apgar scores was 37 weeks. Women with medical diseases or antepartum complications should be more cautious as they have an increased risk of adverse outcomes.

## Supplementary Information

**Additional file 1:** **Supplementary Figure 1.** Incidence of stillbirth by year and gestational age.

**Additional file 2: Supplementary Figure 2. **Incidence of SGA by year and gestational age. 

**Additional file 3:** **Supplementary Figure 3.** Incidence of Low Apgar score by year and gestational age.

**Additional file 4: Supplementary Figure 4. **Association between maternal complications and adverse perinatal outcomes in women of advanced ages.* *Foetuses staying in utero were used as reference in separate gestational ages, women in medical diseases group or antepartum complications group were compared with uncomplicated women. All results were adjusted for the sampling distribution of the population and clustered of births within hospitals and pregnant woman individuals. Covariates were adjusted as area classification, geographic location, hospital level, infants birth year, education, marriage, parity, prenatal examination, twins born sequence and weight imbalance**. ** 

**Additional file 5:** **Supplementary Table 1.**Risk of stillbirth at Each Week of Gestation Compared with Remaining in Utero categorized by maternal complication condition. **Supplementary Table 2.**Risk of SGA at Each Week of Gestation Compared with Remaining in Utero categorized by maternal complication condition. **Supplementary Table 3.**Risk of low apgar score (<4) at Each Week of Gestation Compared with Remaining in Utero categorized by maternal complication condition.

## Data Availability

The datasets generated and/or analysed during the current study are not publicly available due to the terms of our contract with the Chinese National Health Commission but are available from the corresponding author on reasonable request.

## Abbreviations

SGA: Small-for-gestation-age
NMNMSS: National Maternal Near Miss Surveillance System
ITSA: Interrupted Time Series Analysis
GLM: Generalised Linear Model
aORs: Adjusted odds ratios
CIs: Confidence intervals
