# Supplementary figures and images for: Temporal trends and adverse perinatal outcomes of twin pregnancies at differing gestational ages: an observational study from China between 2012–2020
Source: BMC Pregnancy Childbirth. 2022 Jun 3;22:467. doi: 10.1186/s12884-022-04766-0 (PMC9164484; doi:10.1186/s12884-022-04766-0)

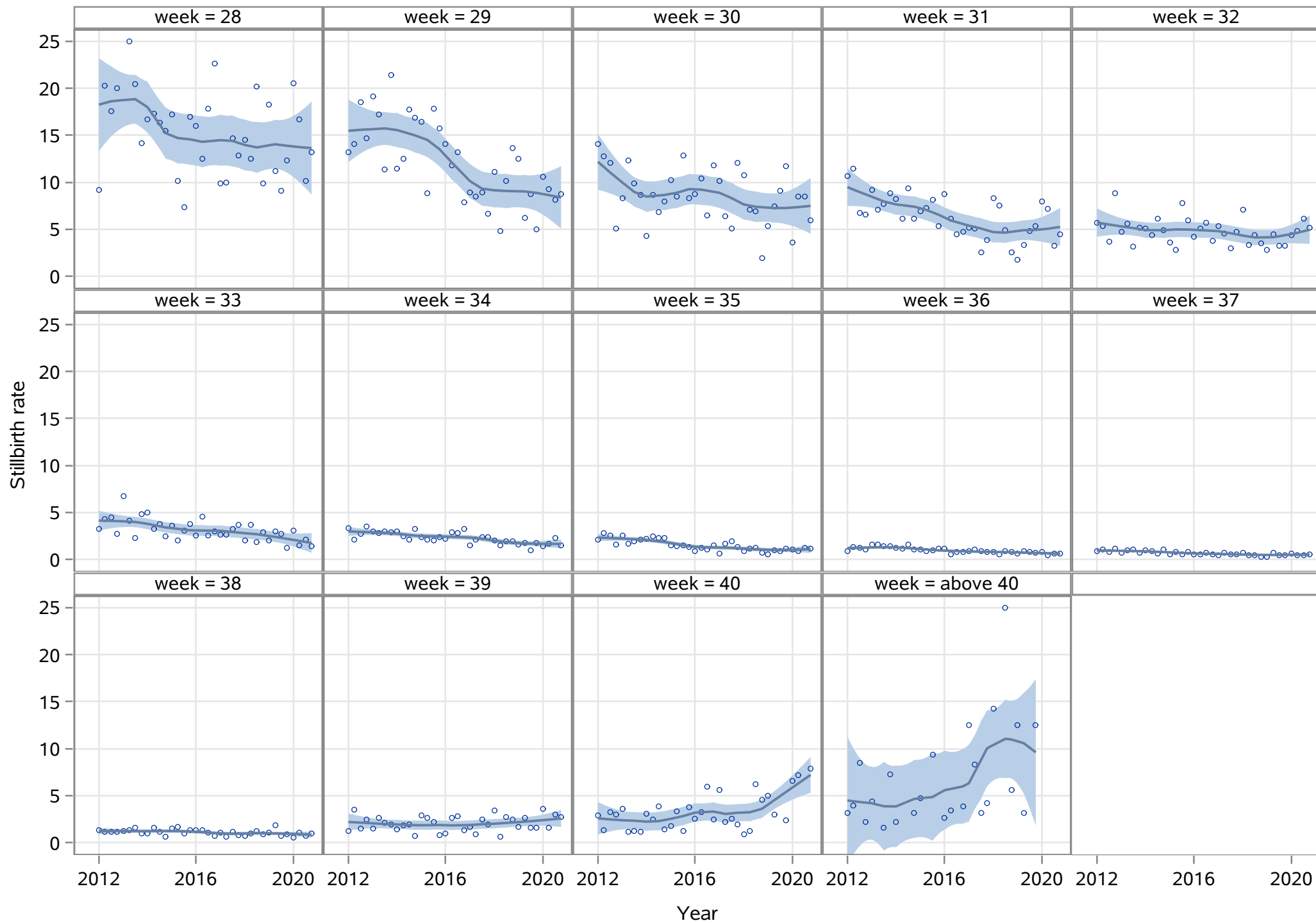

Supplement: Supplementary file 1 — Additional file 1: Supplementary Figure 1. Incidence of stillbirth by year and gestational age. [file 12884_2022_4766_MOESM1_ESM.pdf]

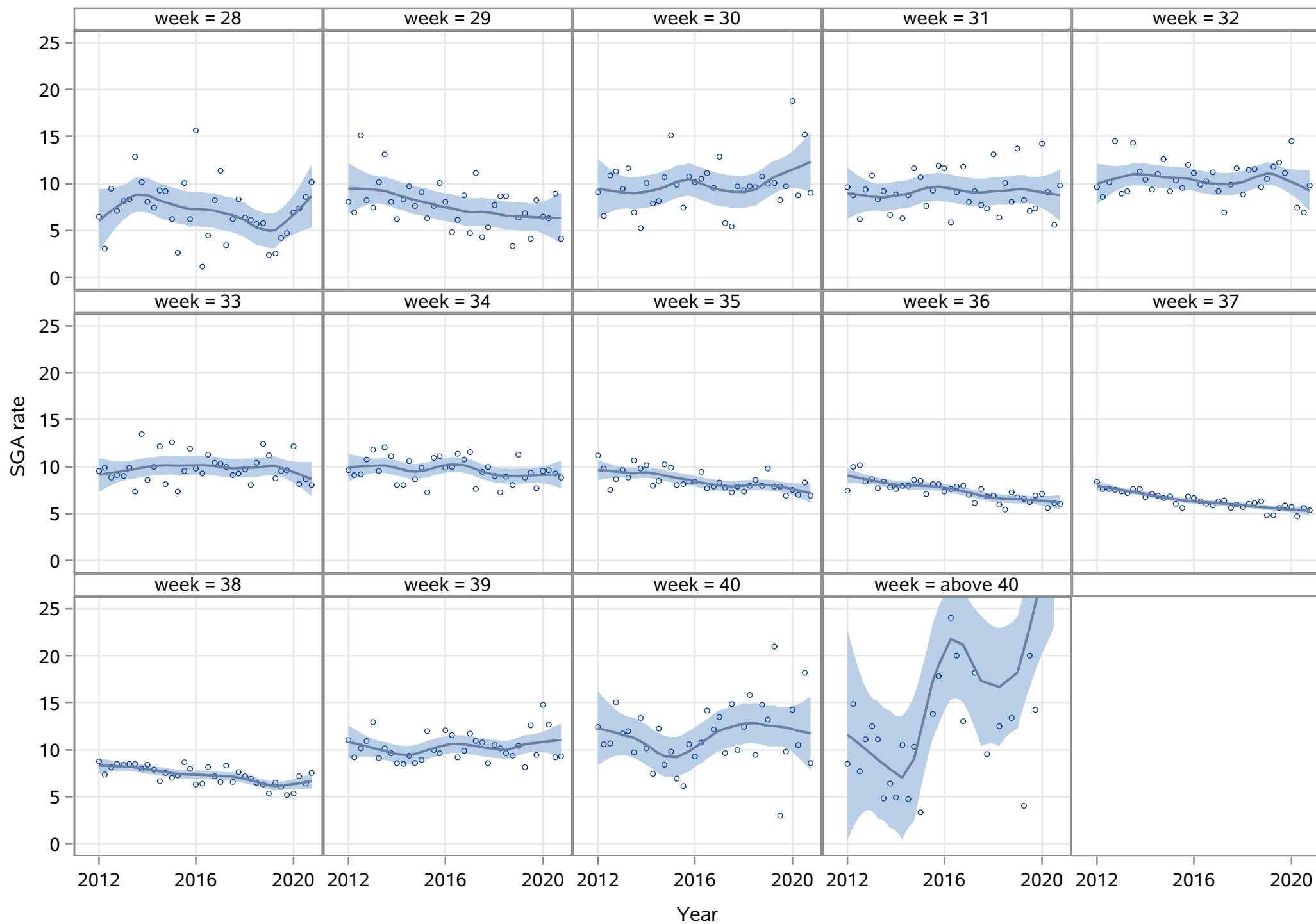

Supplement: Supplementary file 2 — Additional file 2: Supplementary Figure 2. Incidence of SGA by year and gestational age. [file 12884_2022_4766_MOESM2_ESM.pdf]

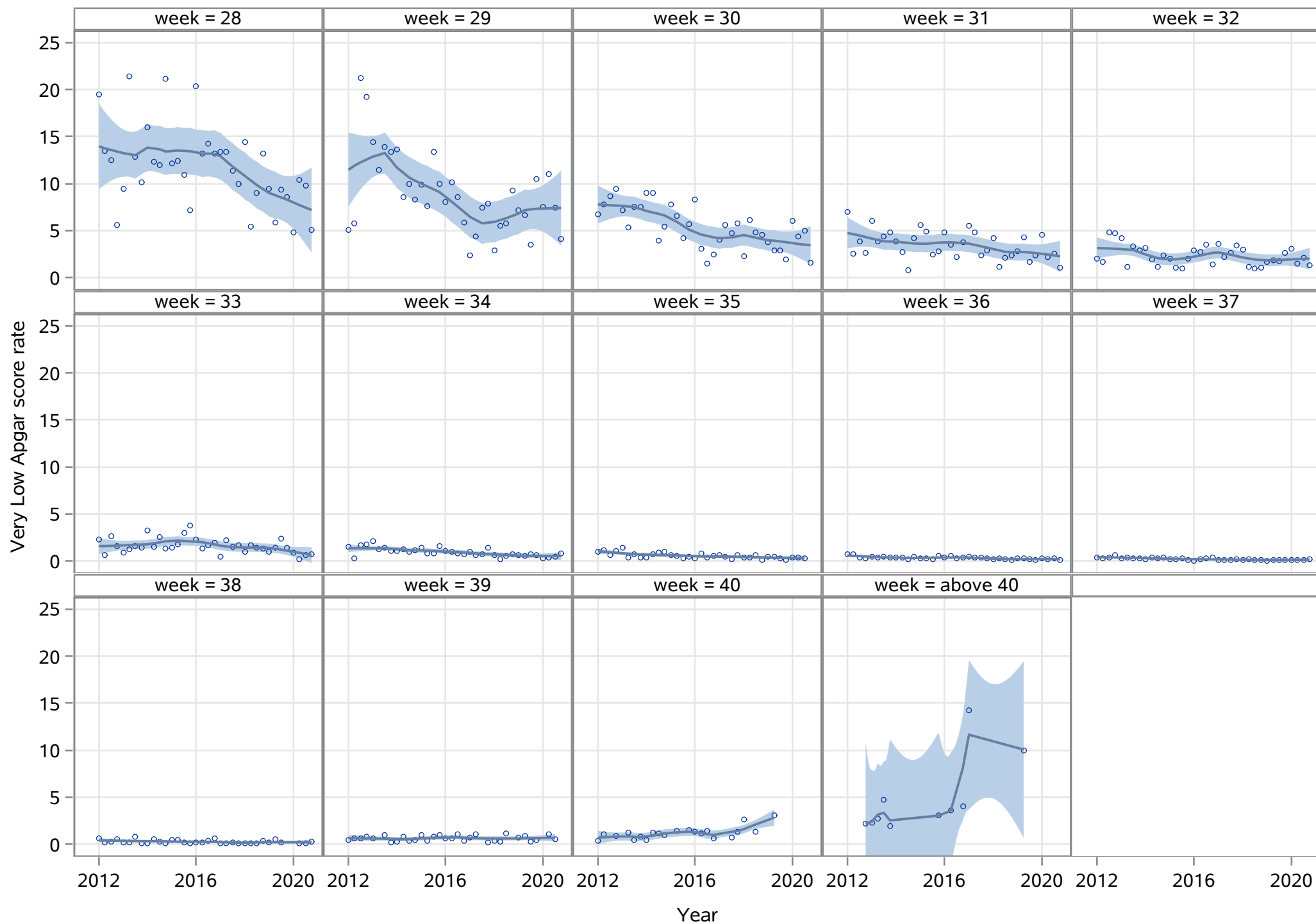

Supplement: Supplementary file 3 — Additional file 3: Supplementary Figure 3. Incidence of Low Apgar score by year and gestational age. [file 12884_2022_4766_MOESM3_ESM.pdf]
