# Supplementary material for: Temporal trends and adverse perinatal outcomes of twin pregnancies at differing gestational ages: an observational study from China between 2012–2020
Source: BMC Pregnancy Childbirth. 2022 Jun 3;22:467. doi: 10.1186/s12884-022-04766-0 (PMC9164484; doi:10.1186/s12884-022-04766-0)
Supplement: Supplementary file 4 — Additional file 4: Supplementary Figure 4. Association between maternal complications and adverse perinatal outcomes in women of advanced ages.* *Foetuses staying in utero were used as reference in separate gestational ages, women in medical diseases group or antepartum complications group were compared with uncomplicated women. All results were adjusted for the sampling distribution of the population and clustered of births within hospitals and pregnant woman individuals. Covariates were adjusted as area classification, geographic location, hospital level, infants birth year, education, marriage, parity, prenatal examination, twins born sequence and weight imbalance. [file 12884_2022_4766_MOESM4_ESM.pdf]

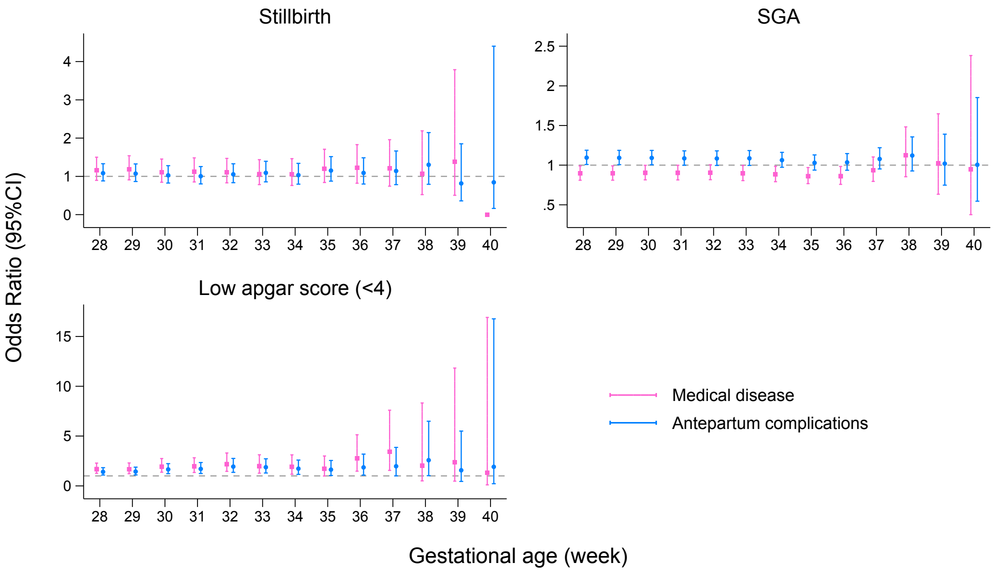

Stillbirth
SGA
Low apgar score (<4)
Odds Ratio (95%CI)
Gestational age (week)
Medical disease
Antepartum complications
0
1
2
3
4
.5
1.5
2.5
5
10
15
28
29
30
31
32
33
34
35
36
37
38
39
40
