# Supplementary material for: Temporal trends and adverse perinatal outcomes of twin pregnancies at differing gestational ages: an observational study from China between 2012–2020
Source: BMC Pregnancy Childbirth. 2022 Jun 3;22:467. doi: 10.1186/s12884-022-04766-0 (PMC9164484; doi:10.1186/s12884-022-04766-0)
Supplement: Supplementary file 5 — Additional file 5: Supplementary Table 1.Risk of stillbirth at Each Week of Gestation Compared with Remaining in Utero categorized by maternal complication condition. Supplementary Table 2.Risk of SGA at Each Week of Gestation Compared with Remaining in Utero categorized by maternal complication condition. Supplementary Table 3.Risk of low apgar score (<4) at Each Week of Gestation Compared with Remaining in Utero categorized by maternal complication condition. [file 12884_2022_4766_MOESM5_ESM.docx]

Supplementary

STable 1. Risk of stillbirth at Each Week of Gestation Compared with Remaining in Utero categorized by maternal complication condition.

| Gestational week | Adjusted OR | | |
| --- | --- | --- | --- |
|  | uncomplicated | Medical disease | Antepartum complications |
| 28 | 13.59[10.21,18.08] | 9.28[6.13,14.05] | 8.53[6.87,10.59] |
| 29 | 6.82[5.17,8.99] | 7.14[4.75,10.75] | 7.31[5.94,8.98] |
| 30 | 5.61[4.27,7.37] | 4.96[3.45,7.11] | 5.29[4.27,6.55] |
| 31 | 4.65[3.63,5.94] | 3.69[2.46,5.53] | 4.21[3.46,5.13] |
| 32 | 2.85[2.23,3.64] | 4.13[2.98,5.73] | 3.31[2.76,3.98] |
| 33 | 2.73[2.19,3.40] | 2.41[1.76,3.29] | 2.54[2.09,3.07] |
| 34 | 2.03[1.62,2.56] | 2.40[1.78,3.23] | 2.15[1.82,2.53] |
| 35 | 1.76[1.45,2.13] | 1.68[1.25,2.25] | 1.51[1.28,1.80] |
| 36 | 1.18[0.98,1.42] | 1.31[0.93,1.84] | 1.04[0.87,1.24] |
| 37 | 0.66[0.53,0.83] | 0.62[0.42,0.93] | 0.58[0.47,0.72] |
| 38 | 0.48[0.36,0.63] | 0.71[0.41,1.22] | 0.67[0.51,0.87] |
| 39 | 0.73[0.48,1.11] | 0.52[0.16,1.67] | 0.57[0.37,0.86] |
| 40 | 0.66[0.30,1.46] | 0.02[0.00,0.29] | 0.53[0.25,1.13] |

All results were adjusted for the sampling distribution of the population and clustered of births within hospitals and pregnant woman individuals. Covariates were adjusted as area classification, geographic location, hospital level, infants birth year, maternal age, education, marriage, parity, prenatal examination, twins born sequence and weight imbalance.

STable 2. Risk of SGA at Each Week of Gestation Compared with Remaining in Utero categorized by maternal complication condition.

| Gestational week | Adjusted OR | | |
| --- | --- | --- | --- |
|  | uncomplicated | Medical disease | Antepartum complications |
| 28 | 0.69[0.50,0.96] | 0.51[0.31,0.82] | 0.79[0.63,1.00] |
| 29 | 0.70[0.54,0.91] | 0.62[0.45,0.86] | 0.89[0.74,1.07] |
| 30 | 1.03[0.87,1.23] | 0.93[0.73,1.18] | 0.96[0.83,1.10] |
| 31 | 0.82[0.70,0.96] | 0.93[0.76,1.15] | 1.01[0.89,1.15] |
| 32 | 1.00[0.87,1.14] | 1.24[1.05,1.47] | 1.14[1.02,1.26] |
| 33 | 1.02[0.91,1.14] | 1.15[1.01,1.32] | 1.09[1.00,1.19] |
| 34 | 1.08[0.99,1.18] | 1.20[1.08,1.33] | 1.18[1.10,1.27] |
| 35 | 1.11[1.03,1.20] | 1.05[0.95,1.16] | 1.11[1.04,1.18] |
| 36 | 1.10[1.03,1.17] | 1.03[0.94,1.13] | 1.03[0.97,1.09] |
| 37 | 0.87[0.81,0.93] | 0.77[0.68,0.88] | 0.89[0.83,0.95] |
| 38 | 0.79[0.71,0.88] | 0.58[0.46,0.72] | 0.78[0.70,0.87] |
| 39 | 0.96[0.81,1.15] | 0.83[0.56,1.23] | 0.87[0.72,1.05] |
| 40 | 0.95[0.63,1.43] | 1.17[0.43,3.20] | 1.23[0.79,1.91] |

All results were adjusted for the sampling distribution of the population and clustered of births within hospitals and pregnant woman individuals. Covariates were adjusted as area classification, geographic location, hospital level, infants birth year, maternal age, education, marriage, parity, prenatal examination, twins born sequence and weight imbalance.

STable 3. Risk of low apgar score (<4) at Each Week of Gestation Compared with Remaining in Utero categorized by maternal complication condition.

| Gestational week | Adjusted OR | | |
| --- | --- | --- | --- |
|  | uncomplicated | Medical disease | Antepartum complications |
| 28 | 21.33[16.07,28.31] | 14.28[9.20,22.17] | 19.18[15.35,23.97] |
| 29 | 16.54[12.51,21.88] | 13.12[8.90,19.33] | 11.88[9.37,15.06] |
| 30 | 10.49[7.73,14.24] | 6.55[4.33,9.93] | 10.07[8.15,12.45] |
| 31 | 6.81[5.06,9.17] | 5.50[3.65,8.29] | 7.56[5.87,9.74] |
| 32 | 5.89[4.38,7.92] | 5.45[3.90,7.61] | 4.11[3.16,5.33] |
| 33 | 3.84[2.87,5.12] | 3.53[2.46,5.06] | 4.00[3.13,5.12] |
| 34 | 2.98[2.18,4.07] | 2.47[1.67,3.66] | 3.56[2.81,4.51] |
| 35 | 2.12[1.57,2.86] | 1.37[0.90,2.09] | 1.90[1.44,2.51] |
| 36 | 1.34[0.97,1.83] | 1.21[0.82,1.78] | 1.07[0.82,1.41] |
| 37 | 0.99[0.67,1.47] | 0.62[0.34,1.12] | 0.79[0.56,1.12] |
| 38 | 0.54[0.33,0.88] | 0.93[0.45,1.95] | 0.41[0.26,0.66] |
| 39 | 0.58[0.29,1.16] | 0.67[0.20,2.23] | 0.49[0.27,0.88] |
| 40 | 0.41[0.15,1.14] | 0.96[0.04,23.22] | 0.74[0.23,2.38] |

All results were adjusted for the sampling distribution of the population and clustered of births within hospitals and pregnant woman individuals. Covariates were adjusted as area classification, geographic location, hospital level, infants birth year, maternal age, education, marriage, parity, prenatal examination, twins born sequence and weight imbalance.
